# Texas Delegate and the International Congress

**Published:** 1887-07

**Authors:** 


### TEXAS DELEGATES AND THE INTERNATIONAL CONGRESS.

We have received many letters of inquiry from physicians about the rail road rates, and about the delegation to the Ninth International Medical Congress, etc., and we give the following information for the benefit of those who contemplate attending.

The Texas State Medical Association, numbering between four and five hundred active members, is entitled to forty odd delegates. The full number, for some reason, was not appointed, and the following are those who have received their credentials from the Secretary's office. In making out the list to be published in the Transactions, the name of Dr. T, D. Wooten was accidentally omitted. The only change that has been made so far as we are informed is, that Dr. F. E. Daniel having declined attending, resigned, and Dr. W. Caston of Corsicana was appointed in his stead.

### DELEGATES.

Drs. Geo. Cupples, B. F. Kingsley and G. G. Watts of San Antonio; Eugene Clark, Lockhart; G. W. Christian, Burnet; A. C. White, Cuero; Jno. C. Jones and R. T. Knox of Gonzales; J. R. Johnson, Bryan; H. A. West and J. F. Y. Paine of Galveston; C. C. Black, Round Rock; R. M. Swearingen, F. E. Daniel, (resigned), J. W. McLaughlin, M. A. Taylor, T. J. Tyner and T. D. Wooten of

Austin; T. H. Nott, Goliad; J. T. Wilson, Sherman; H. H. Darr, Caldwell; T. J. Turpin, Corpus Christi; J. M. Pace, Dallas; D. C. Jones, Calvert; F. R. Martin, Kyle; W. G. Jameson, Rusk, W. T. Evans, Jewett; A. G. Pendleton, San Marcos; J. T. Field, Ft. Worth; I. E. Clark, Moravia; J. W. Carhart, Lampasas; O. Eastland, Wichita; W. Caston, Corsicana.

It is not *necessary* that one should be a delegate from *any* medical organization, or even to be a member of any local or State society, in order to become *a member of the Congress.* Any physician a graduate of a regularly chartered medical college, and in good standing, can become a member of the Congress by making application and paying $10. Of course he has to show his credentials and be endorsed by some one well known; but *delegates,* as we understand it, have, as they do in State Association,—two votes;—members only one.

There is an announcement in the *Jaurnal of the American Medical Association* of July 16, that "all the rail roads have agreed on a uniform rate of one and one-third fare for the round trip, tickets to be issued on the *certificate* plan." The only reduction, or special rate that has been made for Texas, especially, so far as we are informed, is that of the PIEDMONT AIR LINE, from New Orleans, by way of Atlanta, an air line to Washington. In a conversation with the General Agent, J. M. Means, Esq., of Houston, we were authorized to announce that *reduced rates and through* tickets by that line, will be given, on application to him at Houston; and Mr. Means further stated that if there should be a sufficient number going from Texas to justify it, he would give them a handsome palace car from New Orleans, *through, without change,* and would, himself, accompany the party to look after their comfort and accommodation. Mr. Means did not name any amount other than the rate from Austin, which we understood him to say, would be $64.50 for the round trip.

We have not been informed by all who will attend, of those appointed: but Drs. Tyner, Wooten and Taylor of Austin, Dr. Caston of Corsicana, Dr. Eastland of Wichita have declared their intention to attend, and Dr. Wooten has already gone north, and Dr. Tyner will leave on 1st of August. No doubt there will be a full attendance. We hope so, and that Texas may be well and ably represented.

The Journal of the A. M. A. announces that *all the hotels* in Washington have made special rates: and that an excursion to Niagara Falls; one to Mt. Vernon and others, are among the delights planned by the committee of arrangements for the visiting physicians.

